# Reliability and repeatability of 2021 ARCO classification and its guiding significance in treatment of nontraumatic osteonecrosis of the femoral head

**DOI:** 10.1186/s12891-023-06587-4

**Published:** 2023-06-08

**Authors:** Ju’an Yue, Xiaozhong Guo, Randong Wang, Bing Li, Qiang Sun, Wangyan Liu, Jiao Chen, Fengnian Zhao

**Affiliations:** Department of Joint Surgery, Aviation General Hospital, Courtyard 3, Anwai Beiyuan, Chaoyang District, Beijing, China

**Keywords:** Osteonecrosis, Femoral head, ARCO classification, Consistency, Repeatability

## Abstract

**Background:**

The study was designed to evaluate the interobserver reliability and intraobserver repeatability of the 2021 Association Research Circulation Osseous (ARCO) classification and explore its guiding significance in the treatment of nontraumatic osteonecrosis of the femoral head (ONFH).

**Methods:**

In this retrospective study, we randomly selected and investigated 50 sets of preoperative computed tomography or magnetic resonance imaging scans from 96 patients (139 hips) to validate the reliability and repeatability of the 2021 ARCO classification. Patients with a nano-hydroxyapatite/polyamide-66 support rod were included in the clinical efficacy study. The Harris hip score (HHS) was used to assess hip function. Femoral head collapse of > 2 mm was considered radiological failure. Total hip arthroplasty (THA) was performed for clinical failure, and follow-up was discontinued.

**Results:**

The average kappa value of interobserver consistency was 0.652. The average rate of consistency was 90.25%, and the average kappa value of intraobserver consistency was 0.836. Eighty-two patients (122 hips) were enrolled and followed up for a mean of 43.57 ± 9.64 months. There was no significant difference in the HHS among the three groups before surgery, but the difference was statistically significant at the last follow-up. Among them, types 1 and 2 had significantly higher scores at the last follow-up than preoperatively (P < 0.05), whereas type 3 had a lower score at the last follow-up than preoperatively, although the difference was not statistically significant (P > 0.05).According to the imaging evaluation, the failure rate of type 1, 2, and 3 at the last follow-up was 0%, 19%, and 87%, respectively. Univariate analysis showed that the femoral head survival rate of radiography was significantly affected by the new classification system (P = 0.00). At the last follow-up, the incidence rate of THA in type 1, 2, and 3 was 5%, 7%, and 31%, respectively. Univariate analysis showed that the femoral head survival rate was significantly affected by the new classification system (P = 0.001).

**Conclusions:**

The 2021 ARCO classification for early-stage ONFH shows substantial consistency and repeatability. We do not recommend femoral head-preserving surgery for patients with type 3 ONFH.

## Backgroud

Osteonecrosis of the femoral head (ONFH) is a potentially devastating disease [1]. If ONFH is not treated promptly and correctly, it often leads to collapse of the femoral head, which in turn leads to osteoarthritis of the hip [2]. ONFH is common in young adults, and its prevalence is reportedly increasing [3]. In addition, because the disease is more common in young people and the prosthesis has a certain lifespan, young patients are at risk of the need for multiple replacements [4]. Therefore, many scholars have focused on how to improve the success rate of preserving the femoral head in patients with early ONFH [5–7]. Previous studies have shown that the size and location of lesions are important factors affecting the postoperative outcome of early femoral head necrosis [8–10]. Therefore, many studies have attempted to predict the success rate of head preservation surgery in patients with early ONFH by typing the site of the necrotic area [11–14].

In 2021, Association Research Circulation Osseous (ARCO) developed a novel classification system for early-stage ONFH (types 1, 2, and 3) [15] and currently recommends using this method as a unified classification for early-stage ONFH. The present study was designed to evaluate the interobserver reliability and intraobserver repeatability of the 2021 ARCO classification and explore its guiding significance in the treatment of nontraumatic ONFH.

## Materials and methods

This was a retrospective study. All procedures performed were in accordance with the ethical standards of the World Medical Association Declaration of Helsinki Ethical Principles for Medical Research Involving Human Subjects. All methods were carried out in accordance with the Ethics Committee of Aviation General Hospital (No:HK2019-01-04). A total of 96 patients (139 hips) with early-stage ONFH underwent hip-preserving surgery (single approach to double-channel core decompression and bone grafting with structural bone support) from October 2016 to October 2020 in Aviation General Hospital. All patients underwent preoperative anteroposterior and lateral radiographic examinations, computed tomography (CT), and magnetic resonance imaging (MRI) of both hips.

**Fig. 1:**
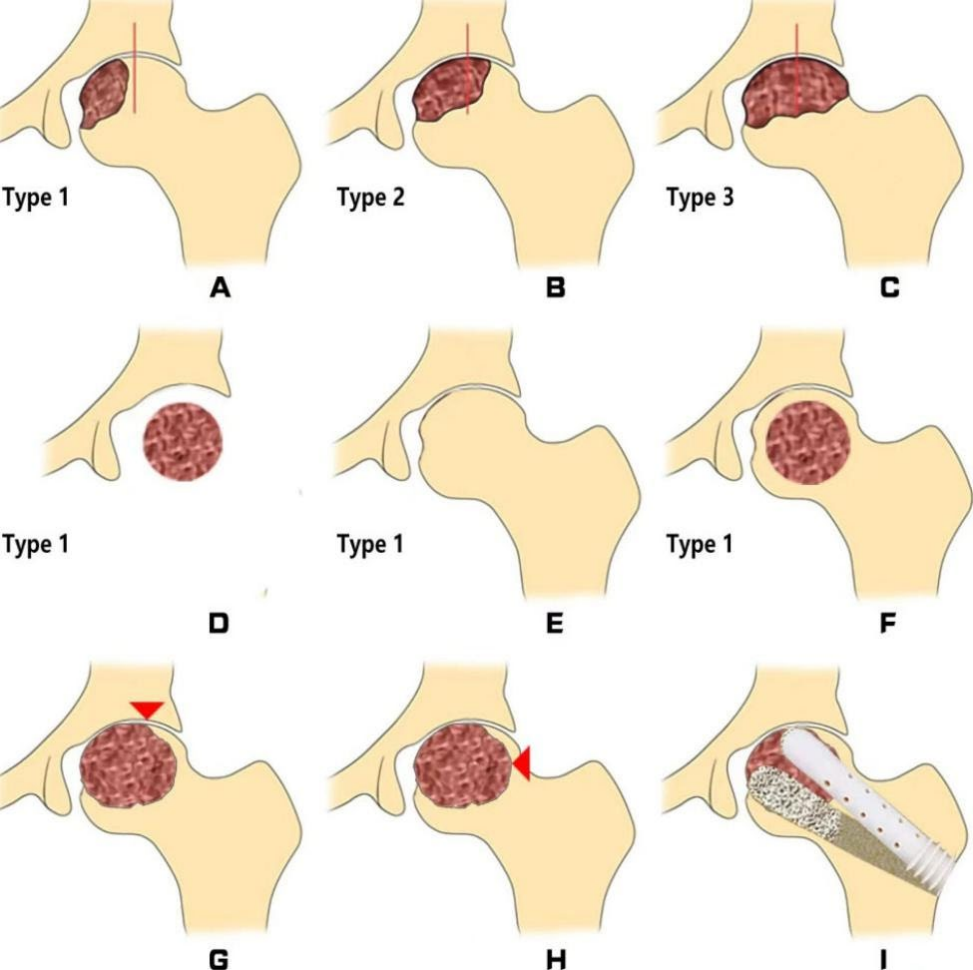
2021 ARCO classification. **(A)** Type 1 is a small lesion confined to the region medial to the apex of the femoral head. **(B)** Type 2 is a medium-sized lesion in which the lateral margin of the necrotic portion is between the apex of the femoral head and the lateral edge of the acetabulum. **(C)** Type 3 is a large lesion that extends laterally to the lateral acetabular edge. **(D, E)** Far anterior lesions that do not appear on mid-coronal images are classified as type 1. **(F)** Intramedullary infarcts that do not involve the subchondral bone are classified as type 1. **(G)** The red arrowhead indicates the correct boundary of necroptosis staging. **(H)** The red arrow head indicates the boundary of frequently misjudged errors. **(I)** Schematic diagram of the operation

The 2021 ARCO classification uses two landmarks: the apex of the femoral head and the lateral edge of the acetabulum [15]. When the lateral margin of the necrotic portion is medial to the femoral head apex, the lesion is categorized as type 1 (Fig. 1A) [15]. When the lateral margin is located between the femoral head apex and the lateral acetabular margin, the lesion is categorized as type 2 (Fig. 1B) [15]. When the lateral margin extends outside the lateral acetabular margin, the lesion is categorized as type 3 (Fig. 1C) [15]. Far anterior lesions (Fig. 1D) that do not appear on mid-coronal images (Fig. 1E) are classified as type 1 [15]. Intramedullary infarcts that do not involve the subchondral bone are also classified as type 1 (Fig. 1F) [15]. In cases of necrosis with geometrical shapes, the lateral border of subchondral necrosis is considered the lateral border of necrosis (Fig. 1G), not the lateral border of the necrotic area (Fig. 1H) [15].

We randomly selected and investigated 50 sets of preoperative CT or MRI scans from 96 patients (139 hips) to validate the reliability and repeatability of the 2021 ARCO classification. According to the protocol of the 2021 ARCO classification, eight residents were recruited and trained to independently perform interobserver reliability and intraobserver repeatability evaluations. Additionally, intraobserver repeatability evaluations were conducted again at 2-week intervals by the same group of residents with presentation of the images in a different order.

Details of the surgical procedures (Fig. 1I) are provided in a previously published article [6]. The Harris hip score (HHS) was used to assess hip function. At each follow-up visit, all patients underwent anteroposterior and frog-leg position radiographs of the hip. while radiographs were used to check the depth of the femoral head collapse. Assessment was based on anteroposterior and lateral radiographs, and femoral head collapse of > 2 mm was considered radiological failure. Total hip arthroplasty (THA) was performed for patients with clinical failure, and follow-up was discontinued.

### Statistical analysis

SPSS version 22.0 (IBM Corp., Armonk, NY, USA) was used for statistical analysis. Data are expressed as mean ± standard deviation. The kappa coefficient was used to determine reliability and repeatability according to the recommendation by Landis and Koch [16]. Specifically, the kappa value ranged from − 1 to 1, and the results were classified as poor (kappa value of < 0.00), slight (0.00–0.20), fair (0.21–0.40), moderate (0.41–0.60), substantial (0.61–0.80), and almost perfect (0.81–1.00). The paired t-test and Wilcoxon test were used to compare the preoperative HHS with the HHS at final follow-up, and between-group comparisons were made using the Kruskal–Wallis test. Rate comparisons were performed using the χ^2^ test. Single risk factor analysis for surgical failure was performed using the Kaplan–Meier method. A P value of < 0.05 was considered statistically significant.

## Results

Data from 50 sets of CT or MRI scans were obtained from 37 patients (left hip, n = 14; right hip, n = 10; both hips, n = 13) with a mean age of 39.59 ± 10.70 years. In total, 2,800 evaluations were performed between different observers, and 400 repeatability evaluations were performed between the same observers. The interobserver reliability consistency among different residents according to the 2021 ARCO classification is shown in Table 1. The average kappa value of interobserver consistency was 0.652 (range, 0.422–0.872). The intraobserver repeatability consistency in the same resident between two performances is shown in Table 2. The average rate of consistency was 90.250%, and the average kappa value of intraobserver consistency was 0.836 (range, 0.680–0.934).

**Table 1:** Interobserver reliability

	Kappa Value Between Different Residents							
Variable	1	2	3	4	5	6	7	8
1	-	0.616	0.703	0.646	0.590	0.528	0.619	0.684
2	0.743	-	0.831	0.835	0.709	0.744	0.806	0.740
3	0.708	0.808	-	0.865	0.606	0.640	0.769	0.769
4	0.719	0.811	0.811	-	0.677	0.616	0.742	0.676
5	0.715	0.683	0.622	0.470	-	0.625	0.622	0.518
6	0.535	0.566	0.564	0.597	0.574	-	0.622	0.553
7	0.552	0.583	0.579	0.550	0.504	0.422	-	0.872
8	0.587	0.681	0.647	0.649	0.506	0.453	0.645	-

**Table 2:** Intraobserver repeatability

Variable	No.of Consistence	Consistency Percentage (%)	Kappa Value
1	45	90	0.810
2	48	96	0.934
3	48	96	0.931
4	44	88	0.809
5	41	82	0.680
6	45	90	0.845
7	43	86	0.774
8	47	94	0.903
Average	45.125	90.250	0.836

At the last follow-up, eight patients (eight hips) were lost to follow-up, and nine patients had traumatic femoral head necrosis. Ultimately, 82 patients (122 hips) were enrolled and followed up for a mean of 43.57 ± 9.64 months. The characteristics of the patients are shown in Table 3. The procedure of the operation is shown in Fig. 1I [17].

**Table 3:** Characteristics of the patients

	Patients	Hips
Male	72	110
Female	10	12
Age(years)	38.59 ± 9.16	-
BMI	25.40 ± 3.48	-
Bilateral	40	80
unilateral	42	42
Alcohol abuse	32	43
Corticosteroid application	38	60
Idiopathic	12	19
Type-1	-	19
Type-2	-	58
Type-3	-	45

Data are presented as mean ± standard deviation or n

BMI, body mass index

**Table 4:** Results of clinical analysis

Type	Harris			Imaging evaluation				THA			
	pre-op	Final follow-up	P	Yes	No	No(%)	Log-rank P	Yes	No	No(%)	Log-rank P
1	82 ± 11	92 ± 4	0.016	19	0	0^AC^	0.000	18	1	5^AC^	0.001
2	79 ± 14	86 ± 2	0.009	47	11	19^AB^	-	54	4	7^AB^	-
3	77 ± 14	71 ± 3	0.091	6	39	87^BC^	-	31	14	31^BC^	-
P	0.311	0.000	-	-	-	0.000	-	-	-	0.002	-

The same letter indicates a statistically significant difference

The mean preoperative HHS was 79 ± 14, and that at the last follow-up was 81 ± 21 (improvement of 3 ± 25) (P = 0.130). Although the HHS at the last follow-up was better than that before surgery, the difference was not statistically significant (P = 0.130). There was no significant difference in the HHS among the three groups before surgery; however, significant differences were found at the last follow-up, with the highest score in type 1, followed by types 2 and 3, respectively. Among them, types 1 and 2 had significantly higher scores at the last follow-up than preoperatively (P < 0.05), whereas type 3 had a lower score at the last follow-up than preoperatively, although the difference was not statistically significant (P > 0.05)(Table 4).

**Fig. 2:**
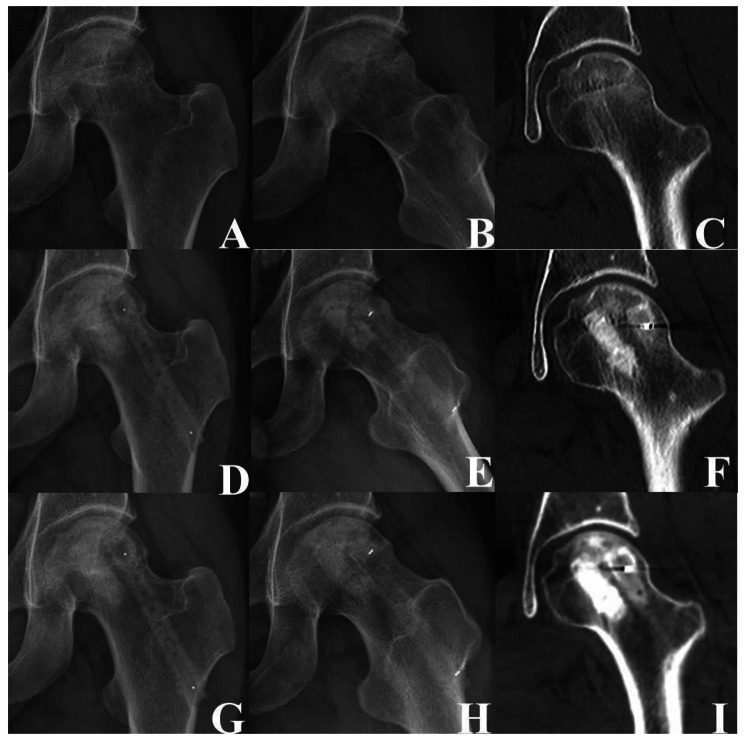
Images of a 36-year-old man who presented with type 1 left idiopathic osteonecrosis of the femoral head. **(A, B)** Preoperative anteroposterior and lateral radiographs showed that the femoral head was round. **(C)** Infarcts were present but did not involve the subchondral bone. **(D–F)** Three days after the operation, radiographs and computed tomography showed the shadow of the support rod and the bone graft. **(G–I)** Three years after the operation, radiographs and computed tomography showed that the femoral head was round and the bone density had increased

**Fig. 3:**
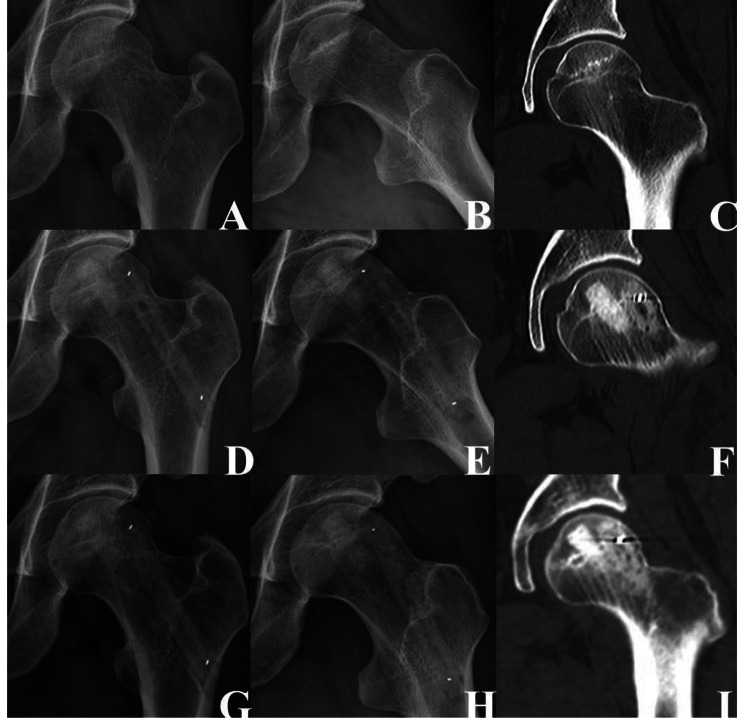
Images of a 50-year-old man who presented with type 2 left nonspecific femoral head necrosis. **(A–C)** Preoperative radiograph and computed tomography showed that the femoral head was rounded. (D–F) Three days after the operation, radiographs and computed tomography showed the shadow of the support rod and the bone graft. **(G–I)** Two years after the operation, radiographs and computed tomography showed that the femoral head was round and the bone density had increased

**Fig. 4:**
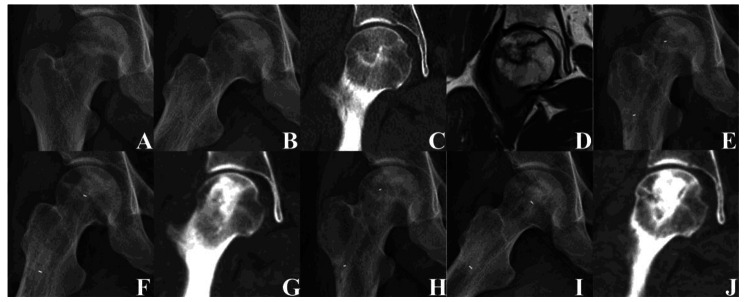
Images of a 30-year-old man with type 3 right steroid-induced osteonecrosis of the femoral head. **(A–D)** Preoperative X-ray and computed tomography showed extensive necrosis of the femoral head with mild collapse. **(E–G)** Three days after the operation, radiographs showed that the femoral head was still round. **(H–J)** Two year after the operation, radiographs and computed tomography showed that the femoral head was round and the bone density had increased

**Fig. 5:**
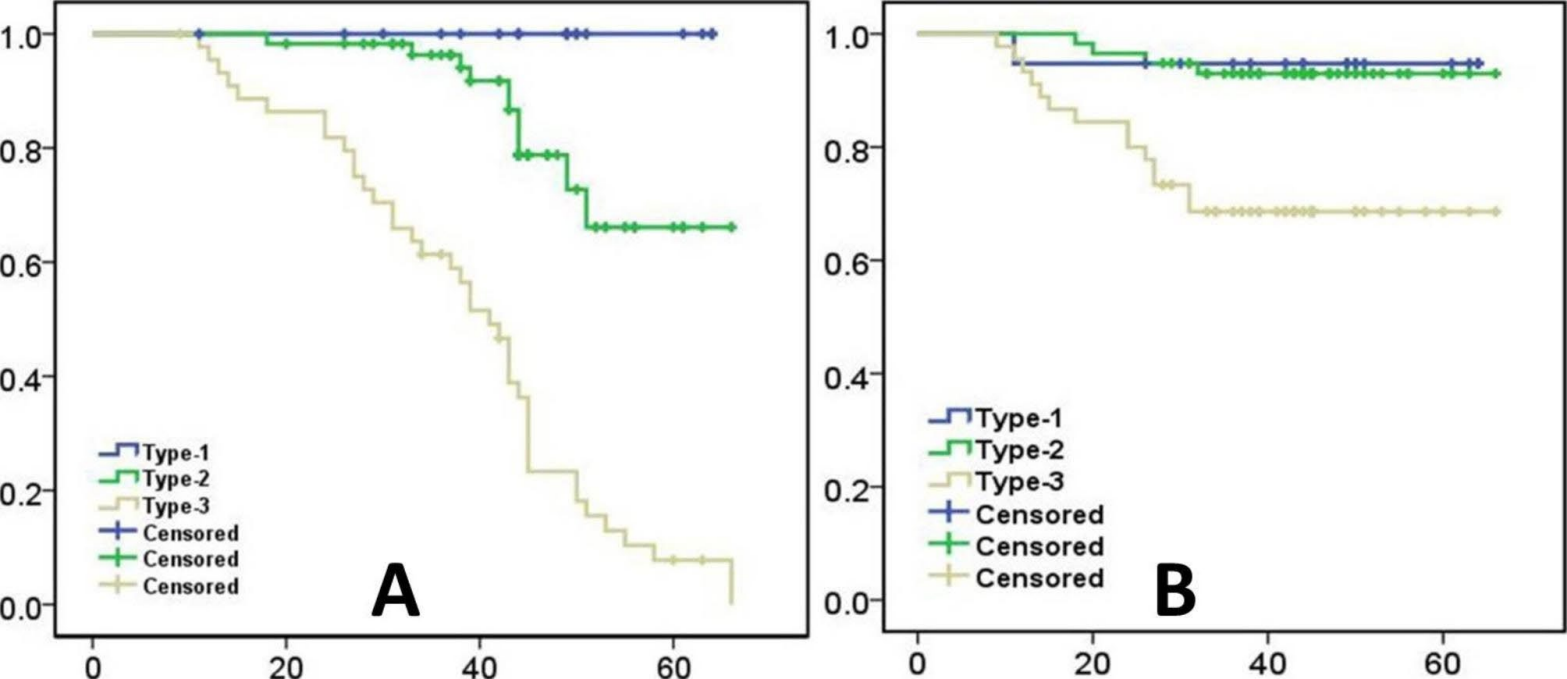
Kaplan–Meier survival curves. **(A)** Stratified according to the imaging changes. **(B)** Stratified according to the endpoint of revision to THA

According to the imaging evaluation, the failure rate of type 1 (Fig. 2), type 2 (Fig. 3), and type 3 (Fig. 4) at the last follow-up was 0% (Success rate: 100%), 19% (Success rate: 82%), and 87% (Success rate: 13%), respectively. The difference in the failure rate among the three groups was statistically significant (P < 0.05). The failure rate of type 2 was significantly higher than that of type 1 (P < 0.05), and that of type 3 was significantly higher than that of types 1 and 2. The univariate analysis showed that the femoral head survival rate was significantly affected by the new ARCO classification system (P = 0.00) (Fig. 5A) (Table 4).

At the last follow-up, the estimated incidence rate of THA in patients with type 1 ONFH was 5%, that in patients with type 2 was 7%, and that in patients with type 3 was 31%. The rate of THA was significantly different among the three groups (P < 0.05). The failure rate of type 2 was significantly higher than that of type 1 (P < 0.05), and that of type 3 was significantly higher than that of types 1 and 2. The univariate analysis showed that the femoral head survival rate was significantly affected by the new ARCO classification system (P = 0.001) (Fig. 5B) (Table 4).

## Discussion

ONFH is associated with a high disability rate. Most affected patients are young; however, the long-term effect of THA is uncertain in young people [18, 19]. Therefore, hip preservation therapy is an important method to delay progression of the disease and has been widely studied in recent years [1, 17]. Once ONFH is diagnosed, it should be staged [20]. The purpose of staging is to guide the formulation of a treatment plan, judge the prognosis, and evaluate the treatment efficacy [21]. Thus, accurate evaluation and classification of the size and location of the necrotic area are of utmost importance [15]. Three classification systems are currently in widespread use: the Steinberg classification, the Japanese Investigation Committee classification, and the modified Kerboul classification. However, all three have limitations [13, 22–25]. Thus, ARCO developed a novel classification system for early-stage ONFH in 2021, and the classification is a highly reliable and valid method [15].

An effective staging method should be simple, effective, reproducible, and clinically instructive. In this study, images were randomly selected and observed by new training residents to determine the interobserver and intraobserver consistency. The average kappa value of interobserver consistency was 0.652, and that of intraobserver consistency was 0.836. According to the standards of Landis and Koch [16], the results showed that 2021 ARCO classification had a substantial degree of consistency and repeatability. We obtained the following four findings through clinical observation. (1) At the last follow-up, the HHS of type 1 and 2 ONFH were significantly higher than those before the operation. Although there was no significant difference in the HHS of type 3 between the preoperative period and the last follow-up, we found that the score tended to decrease after surgery. (2) Type 3 had the highest imaging failure rate at 87%. (3) The rate of THA was also highest in type 3. (4) The single risk factor analysis showed that the 2021 ARCO classification was helpful in predicting the outcome of imaging progression and the incidence of THA.

Koo et al. [15] only observed the imaging changes at different stages of ONFH. The present study is the first to explore the guiding significance of the new staging classification in predicting clinical efficacy after surgery through clinical observation, and it showed that the postoperative efficacy was poor in patients with stage 3. We found that although patients in stage 1 showed no disease progression on imaging, one patient (one hip) underwent THA because of dysfunction. Therefore, the relationship between hip function and imaging needs further study.

The present study has some limitations. First, the surgical outcomes are from a single center. Second, the sample size was small and the follow-up time short. Large sample and long-term follow-up results are thus needed.

## Conclusion

The 2021 ARCO classification for early-stage ONFH shows a substantial degree of consistency and repeatability. The new classification can predict the curative effect after femoral head-preserving surgery. Therefore, we do not recommend femoral head-preserving surgery for patients with type 3 ONFH. Future confirmatory studies with larger and different patient cohorts are warranted.

## Data Availability

All data and materials used to support the findings of this study are included within the article.

## Abbreviations

ONFH: Osteonecrosis of the femoral head
ARCO: Association Research Circulation Osseous
CT: Computed tomography
MRI: Magnetic resonance imaging
HHS: Harris hip score
THA: Total hip arthroplasty
